# Facilitation of Vaginal Delivery in an Infant with Complete Heart Block Secondary to Maternal Anti-Ro Antibodies

**DOI:** 10.1155/2017/8352320

**Published:** 2017-11-21

**Authors:** E. Thornton, L. Tripathi, S. Shebani, I. Bruce, L. Byrd

**Affiliations:** ^1^ST1 Obstetrics and Gynaecology, Royal Bolton Hospital, Bolton, UK; ^2^North Manchester General Hospital, Manchester, UK; ^3^Glenfield Hospital, Leicester, UK; ^4^Department of Rheumatology, Manchester Royal Infirmary, Manchester, UK; ^5^St Mary's Hospital, Manchester, UK

## Abstract

Congenital heart block (CHB) is a rare disorder that may be associated with a high morbidity and even mortality, with a risk of death both in utero and during infancy. Women with serum titres of anti-Ro and/or anti-La antibodies carry a risk of CHB of 1–5% in their offspring, with a recurrence risk of approximately 20%. We present a case of a 36-year-old female with a pregnancy complicated by congenital heart block. Autoimmune profiling at booking showed she was positive for lupus anticoagulant and anti-Ro antibodies. A fetal echocardiogram at 21 + 3 showed complete heart block. She was monitored throughout the remainder of her pregnancy with serial growth scans, cardiovascular profiling, and BPP scoring. She had a normal vaginal delivery at term to a female infant.

## 1. Background

Congenital heart block (CHB) is a rare disorder with an incidence of 1 in 22,000 live births [1]. It may be associated with high morbidity and even mortality (15–30%) with a risk of death both in utero and during infancy, particularly if the heart is structurally abnormal. As many as 67% of survivors require permanent cardiac pacing. Women with serum titres of anti-Ro and/or anti-La antibodies carry a risk of CHB of 1–5% in their offspring, with a recurrence risk of approximately 20%. The condition carries not only neonatal risks but a burden of maternal risk with approximately 50% of mothers developing obstetric complications [2].

## 2. Case Report

Mrs. N is a 36-year-old with G 4 P3 booked at 7-week gestation and viability and EDD of 10/3/10 confirmed by ultrasound scan. She had a history of Sjogren's Syndrome and postpartum DVT and was positive for lupus anticoagulant and anti-Ro antibodies. This pregnancy was managed in the multidisciplinary joint Obstetric and Haematology Clinic and Low Molecular Weight Heparin (LMWH) was commenced at presentation.

**Figure 1:**
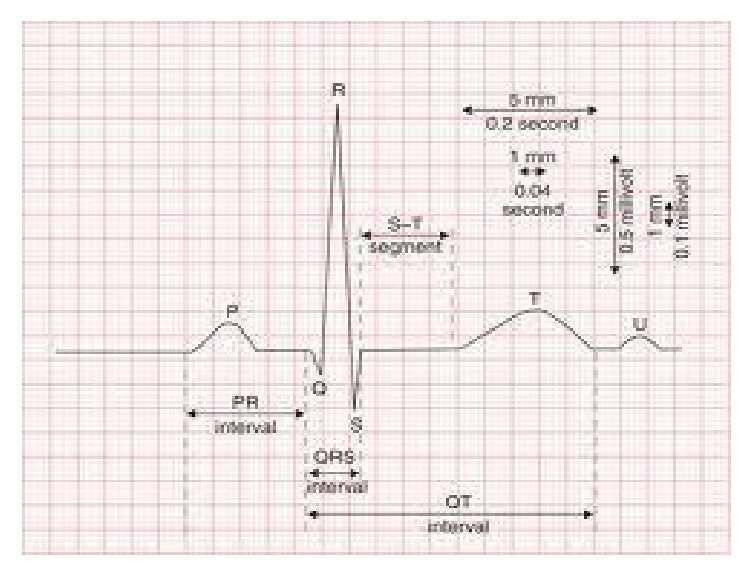
Normal electrical PR (ePR) interval on surface ECG as a guide.

**Figure 2:**
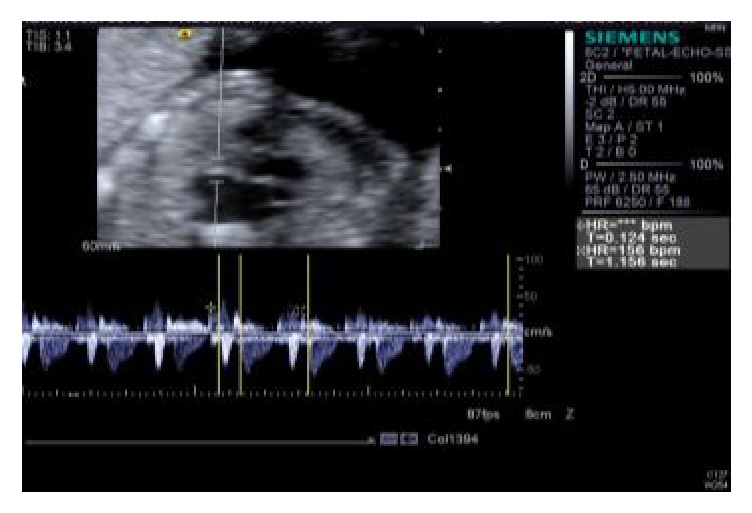
Normal mechanical PR interval (mPR) 10 days earlier in our case; fetal cardiac Doppler sampling the mitral inflow to aortic outflow.

**Figure 3:**
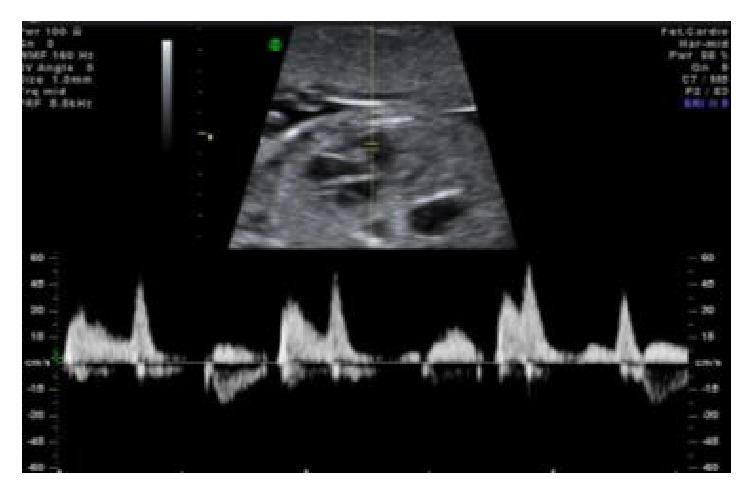
Complete heart block.

An anomaly scan at 20 weeks was unremarkable and a fetal echocardiogram confirmed a structurally normal heart with a normal PR interval (see Figures 1 and 2). Unfortunately 10 days later a repeat scan confirmed complete heart block, with a ventricular rate of 65 bpm (see Figure 3). There were no fetal hydrops or any evidence of heart failure. The couple was counselled regarding the potentially poor outcome and possible risk of fetal death in utero. A plan was agreed to assess fetal cardiac function every week throughout the remainder of the pregnancy. Dexamethasone was given (4 mg daily) the following week as the scan suggested incomplete 2 : 1 block. However, two weeks later complete heart block was reconfirmed and dexamethasone was then discontinued. Its use would however be reconsidered if cardiac failure ensued.

**Figure 4:**
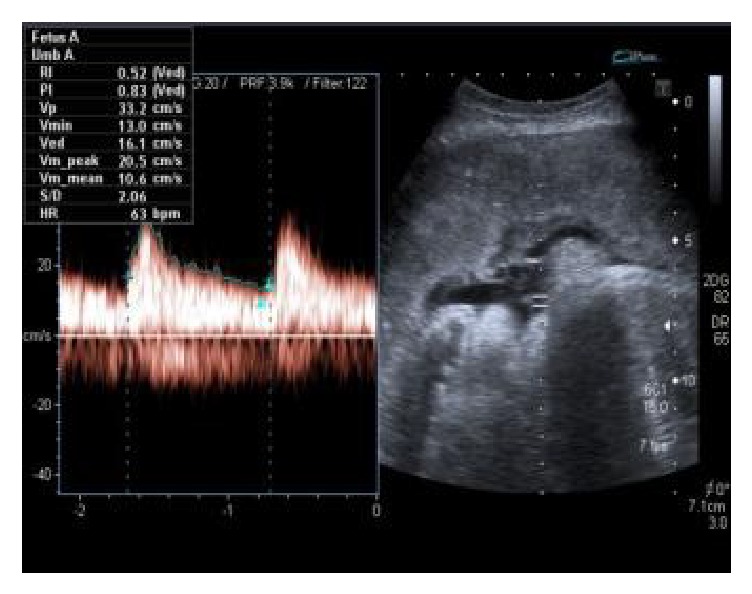
(Umbilical artery Doppler. Good EDF, PI 0.83).

**Figure 5:**
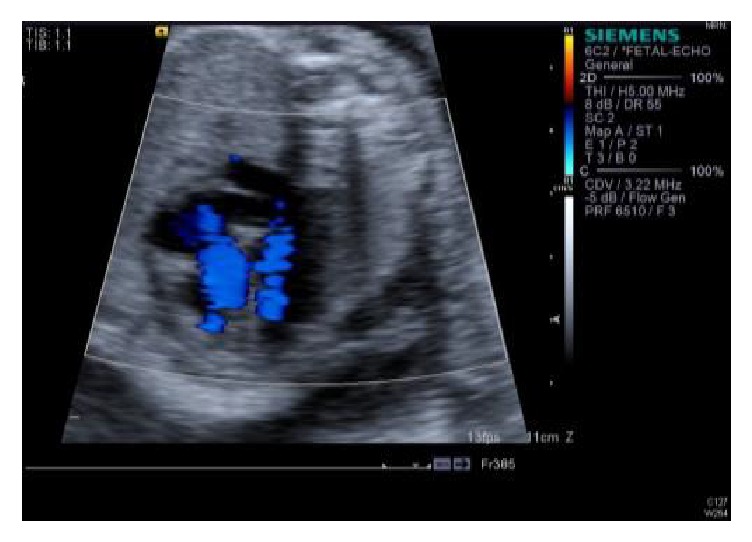
(No atrioventricular regurgitation. No cardiomegaly).

**Figure 6:**
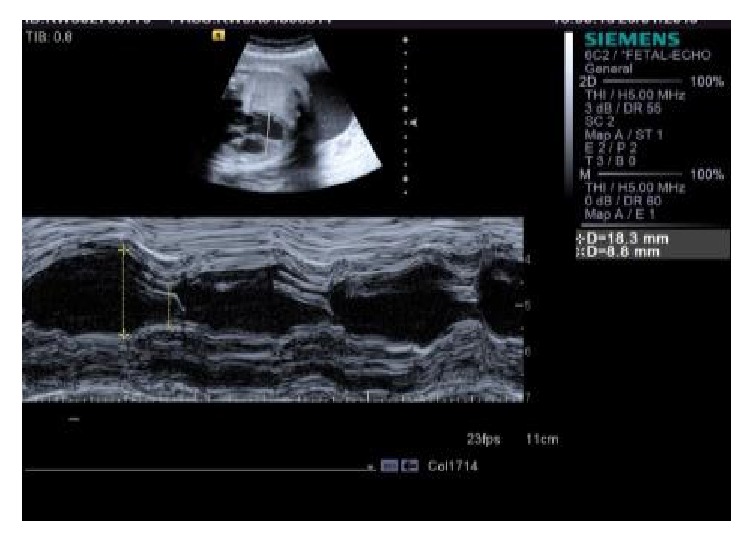
Preserved ventricular contractility (indicated by Fractional Shortening of 52%).

Mode of delivery was initially discussed at 28 weeks and agreed at 36 weeks gestation. Given her parity and history of quick labours the aim was to avoid surgery. Serial growth scans confirmed a fetal AC on the 50th centile, with good growth velocity. Umbilical artery Doppler showed good end diastolic flow and a pulsatility index of 0.83 (Figure 4). Cardiac function, CVP score (see discussion), was maintained throughout (Figures 5 and 6), with a ventricular rate between 65 and 75 bpm. Biophysical Profile (BPP) was assessed from 36 weeks. Good fetal movements were regularly reported by mum. Intermittent auscultation +/− FBS if required, was to be used in labour.

At term a membrane sweep was offered and Mrs. N was admitted to hospital (at her request) 24 hours later with mild tightening. A BPP during the latent phase of labour was reassuring. Labour established later the same day, progressing rapidly to a spontaneous vaginal delivery of a female infant weighing 3.02 kg with Apgars of 9 at 1 and 5 minutes. She was transferred to SCBU for assessment and observation. An ECG confirmed complete heart block, and an echocardiogram reaffirmed good cardiac function despite a ventricular rate of 55 bpm. She established demand feeding over the next 48–72 hours without compromise and was discharged home with mum within the week. Although she remains under review, at the time of Mrs. N's postnatal review in the JOHC her daughter was thriving and had not required cardiac pacing.

## 3. Discussion

At approximately 12 weeks of gestation maternal IgG antibodies against Ro and La intracellular ribonuclear protein are* actively transferred* across the placenta. These are thought to bind to specific cells within the fetal conducting system, resulting in inflammation, scarring, and/or fibrosis. With this in mind Saxena et al. (2015) [3] showed an elevation in cord blood inflammatory markers including CRP and NT-ProBNP in infants who subsequently developed neonatal lupus syndrome.

Whilst CHB is the main cardiac manifestation in infants exposed to anti-Ro/La antibodies other pathologies (to include cardiomyopathy and/or valvular heart disease) also occur [4].

Mortality from heart disease unfortunately remains high with the majority of deaths occurring in utero or early infancy [5] secondary to CHB [6]. Risk factors for poor outcome include presence of structural heart disease, hydrops fetalis, low heart rate (<55 bpm), prematurity, and/or male gender.

Whilst fluorinated steroids, like dexamethasone, can cross the placenta and may treat first or second degree heart block, they have no benefit in third degree heart block which is considered complete and irreversible [7]. A secondary role in improving cardiac function in those fetuses in utero with heart failure has also been considered [8] but robust data is lacking and therefore a pragmatic approach is required as maternal use of dexamethasone is, of course, not without risks. These include the glucocorticoid associated risks of increased infection, loss of bone density, diabetes, hypertension, and cataracts. The fetal risks of maternal steroids include oligohydramnios, intrauterine growth retardation, and adrenal suppression. There is also some suggestion of a risk to the developing fetal brain when exposed to steroids [9].

Based on the assumption that treatment for identified heart block in utero may be effective if it can reduce a generalized inflammatory insult and lower the titre of maternal autoantibodies, several prenatal therapeutic protocols have been utilized. All women at risk with antibodies present should be closely followed during the pregnancy with serial echocardiograms, specifically looking for the earliest signs of conduction system disease such as PR interval prolongation by Doppler (see later discussion). If complete heart block is recently diagnosed (within three weeks of onset), a therapeutic course of dexamethasone 4 mg orally once a day may be tried.

Prenatal dexamethasone may have a role in preventing late onset cardiomyopathy, which frequently requires pacing; however, this viewpoint is far from universal [10]. By contrast a more consistent preventative approach is the use of maternal hydroxychloroquine in high risk pregnancies, though further study is required [11].

It has hitherto been suggested that all women at risk of fetal cardiac disease because of a previous history and/or high positive antibodies should be followed up with serial echocardiograms specifically looking for any prolongation in the PR interval. Nevertheless it has subsequently been established that the onset of CHB is far from predictable and measurement of the mechanical PR interval lacks sensitivity (44%) and/or specificity (88%). Whilst it may be useful in diagnosis, it has no role in prediction or prognosis. However, the electrical PR interval (whilst not available at the time of this case) is proving to be more useful (sensitivity 66%, specificity 96% [12]).

In our patient, Mrs. N, the changes in the degree of heart block was sudden and unpredictable. However, regular/frequent surveillance permitted prompt recognition which was then amenable to steroid therapy; despite this no benefit was achieved as therapy did not reverse the heart block. This agrees with outcome data from larger data sets. Prenatal dexamethasone would appear to have a role in the prevention of cardiomyopathy necessitating frequent cardiac pacing.

For those infants that survive pregnancy the main dilemma is then how to deliver. Continuous CTG is not possible and there is concern that the fetal heart in heart block does not have the capacity to withstand the demands of labour. The BPP is a 10-point fetal assessment tool first described by Manning et al. [9] in 1980. It evaluates fetal breathing, movement, tone, reactivity, and amniotic fluid volume and has been shown to correlate with fetal asphyxia, acidosis, and poor outcome. However, as the most sensitive marker of acidosis is the heart rate component which clearly cannot be assessed in cases of CHB, the BPP is then less than ideal in this situation. With this in mind Donofrio et al. [13] in 2004 utilised a Cardiovascular Profile (CVP) score, originally described by Huhta in 2001 [14] to assess cardiac function, since its introduction CVP scoring has also become valuable in evaluating the prognosis of hydrops when seen on echocardiography [15]. The score subtracts points for the presence of abnormal signs to include hydrops (late sign), venous pulsations, umbilical artery Doppler, cardiac enlargement, and atrioventricular valve regurgitation. To be used effectively the CVP score should be interpreted in consideration with the underlying disease pathology, for example, distinguishing between overload, myocardial disease, and arrhythmias [16]. A similar approach was adopted here. In CVP assessment in CHB then

- umbilical venous pulsation is only scored when noted to be at the atrial rate;
- intermittent holosystolic tricuspid and/or mitral regurgitation is normal in CHB and therefore not scored;
- diastolic AV valve regurgitation is low, common, and not scored;
- small effusion can be seen secondary to inflammation and should therefore be scored.

Excellent cardiac function was maintained throughout pregnancy (CVP score 10/10) and vaginal delivery, at term, of a female infant, was achieved.

## 4. Conclusion

With the advances in the ultrasound technology the prenatal diagnosis of autoimmune CHB has become standard care in most institutions. Fetal echocardiogram with measurement of the mechanical PR interval would not predict but will allow earlier diagnosis of heart block and/or the possibility of very early treatment, which may be able to reverse the disease [12].

On the other hand electrical PR reproducibility, sensitivity, and specificity are superior, which, if available, would be the diagnostic tool of choice to use in any trial to investigate both the natural history and therapy of conduction abnormalities in Ro/La pregnancies.

What really made a difference is that this case was the consistently reassuring high CVP score through cardiothoracic ratio, atrioventricular valve regurgitation fraction shortening, absence of hydrops, and normal fetal umbilical Doppler.
